# ‘Jack be nimble, Jack be quick…’: mental health and psychosocial response in the time of coronavirus

**DOI:** 10.1017/gmh.2020.15

**Published:** 2020-08-05

**Authors:** Stephanie L. Smith, Giuseppe J. Raviola

**Affiliations:** 1 Partners In Health, 800 Boylston Street, Suite 300, Boston, MA 02199, USA; 2 Department of Psychiatry, Brigham and Women's Hospital, 75 Francis Street, Boston, MA 02115, USA; 3 Department of Psychiatry, Massachusetts General Hospital, 55 Fruit Street, Boston, MA 02114, USA

The novel coronavirus pandemic has the potential to disrupt global human development like no other disaster since the Second World War. Alongside the massive bio-social and economic impact of the coronavirus disease 2019 (Covid-19) crisis, the mental health burdens caused by infection-related fears, social distancing, isolation and quarantine, and illness, as well as prolonged economic concern, may be substantial and persist long after the pandemic ends. Youth, those living with preexisting mental health conditions, people in migration, the homeless, and older adults across the globe will be particularly vulnerable to emotional and social disruption wrought by Covid-19.

Although the full mental health and psychosocial impact of the Covid-19 crisis is yet unknown, mental health systems and programs across the globe have an obligation to act quickly to help mitigate the outbreak in whatever ways they can, and especially to work to mitigate the long-term mental health impact on service users and broader populations. Effective mental health and psychosocial response to Covid-19 will also help health systems to improve mental health services, and ‘build back better’ from this crisis, for the future (Epping-Jordan *et al*., 2015). To respond effectively now, systems and their mental health programs must be nimble in adapting existing practices to aggressively address current and evolving mental health needs in the context of the rapid social change and the significant social and moral distress across governments, economies, hospitals, and communities caused by the coronavirus pandemic.

An effective mental health and psychosocial response to any humanitarian or other crisis should ideally encompass several key components: a rapid and efficient *needs assessment*; robust *service and care delivery* adaptations that optimize digital tools and takes advantage of various innovative communication technologies; lean and nimble *program management*; *training and sustained supervision* of clinical work; meaningful *data collection* with monitoring and evaluation; and *analytic and academic work to disseminate innovative interventions*. These components in turn rely upon several core building blocks, including: the contextual adaptation of international standards of mental health and psychosocial care and support in Covid-19 pandemic response and humanitarian crisis response (such as Interagency Standing Committee (IASC) Guidelines (Inter-Agency Standing Committee; Inter-Agency Standing Committee (IASC); Inter-Agency Standing Committee (IASC)) and Psychological First Aid (PFA) (World Health Organization, War Trauma Foundation and World Vision International 2011); mental health service delivery guidelines (such as the World Health Organization's Mental Health Gap Action Program (World Health Organization Mental Health Gap Action Program); knowledge of effective clinical services and training systems design, including quality improvement methodology; the application of key social medicine and anthropology concepts to deepen understanding of the nature and impacts of rapid social change globally; and meaningful and innovative use of lessons learned from the field of global mental health on the mobilization of non-specialists (‘task-sharing’), self-care, and peer support to address the burden of mental and emotional distress and disorders across populations.

More specific to the current moment, to help to prevent and then respond to the mental health burdens catalyzed by the current crisis, mental health systems and psychosocial programs must mount both Covid-19 specific responses as well as quickly adapt mental health service delivery to care settings where infection prevention and control is paramount. Front-line managers and mental health service organizers implementing Covid-19 responses require rapid operational recommendations to guide safe and context-appropriate mental health service and program adaptations to address this unprecedented challenge to effective mental health care delivery. The Mental Health team at Partners In Health (PIH), a non-profit health delivery strengthening organization operating in 10 countries, has worked rapidly to support local mental health teams to prepare and respond to the Covid-19 crisis. We here describe core dimensions to our integrated planning for both pandemic-related mental health and psychosocial response and mental health service adaptations.

We have initiated immediate regular clear communication channels with each mental health manager and clinical team at each country site, using preexisting established communication networks including weekly video and teleconferencing, WhatsApp groups, and other digital platforms. We have also escalated the frequency and expanded the scope of an established monthly learning collaborative designed for the ongoing sharing of mental health lessons among programs and clinicians throughout the PIH network, to include Covid-19 crisis response. A cross-site tracker has been created to share daily updates and lessons learned across country sites.

Our core shared strategy for our mental health and psychosocial crisis response and service adaptation is aligned with, and supports the overarching PIH Covid-19 response strategy, available at https://www.pih.org/article/pihs-emergency-coronavirus-response. Our mental health and psychosocial response prioritizes (1) limiting the spread of Covid-19; (2) sustaining crucial ongoing mental health services and reducing impact on those already vulnerable due to mental health conditions; and (3) reducing or mitigating the mental health suffering and improving functioning of populations affected by Covid-19.

We are using a previously developed PIH cross-site mental health service delivery value chain (Mental Health at Partners In Health (PIH), 2019) as well as the IASC Guidelines in humanitarian settings to identify which service delivery activities during the Covid-19 crisis are essential to maintain throughout the pandemic (Table 1).

**Table 1.** Essential mental health and psychosocial service delivery activities during the Covid-19 crisis

Community- and facility-based crisis management and referral pathways for mental health emergencies
Psychoeducation and basic psychological support focused on the current crisis, including PFA
Medication management for current service users
Pro-active case management of most vulnerable service users
Continued psychotherapy for those with significant functional impairments
Maintaining regular clinical supervision and support of non-specialist providers performing crucial mental health service-related activities
Food and other social support for most vulnerable service users

Across PIH sites, we are adapting these crucial mental health clinical and service activities to limit the spread of Covid-19. Our adaptations include: minimizing time spent by patients at health care facilities by increasing the duration between visits especially for stable patients; providing several months of medications to patients as feasible; establishing home delivery mechanisms for medications as well as guidelines for safe home delivery using personal protective equipment in collaboration with the PIH community health and non-communicable disease (NCD) teams; spreading out waiting area seating and moving waiting areas outside as feasible; setting up handwashing stations for all staff and patients at entrances to mental health clinics; and replacing in-person visits (including formal psychotherapy delivered by non-specialist providers) with telephone visits as feasible. Clinical supervision and support has transitioned entirely to remote supervision via telephone or videoconferencing.

We have developed proactive outreach activities for both active mental health service users as well as vulnerable population groups including the elderly, pregnant women, children, and those at risk of domestic violence. Lists of service users at high risk for psychiatric decompensation, self-injury, relapse or other safety concerns have been created, and direct outreach via telephone or home visits are ongoing, to develop safety plans with service users and families in the context of quarantine or positive Covid-19 testing, including supporting people with needs such as food, soap and other basic necessities. PFA, delivered primarily by non-specialist providers, is offered to individuals in quarantine or isolation. In collaboration with the Commonwealth of Massachusetts (United States), PIH has deployed hundreds of non-specialist contact tracers to reach contacts of those testing positive for Covid-19, refer them for testing and quarantine as necessary, provide PFA, and connect to needed social support throughout their quarantine. Contact tracers also provide individuals with referral to mental health resources as needed. Formal peer support mechanisms for health and other front-line workers have been created and implemented across all PIH sites, including the new Massachusetts Community Tracing Collaborative. Initiatives such as this one introduce the possibility of integrating best practices from experience in global mental health delivery to the US context, including the adaptation of basic packages of psychological care delivered by non-specialist providers. At all PIH global sites, outreach activities – including educational messaging around Covid-19, general coping strategies, and where to find help during the crisis – are shared with populations using text messaging, radio, and social media. We have also reached out to other organizations engaged in similar work, to promote partnerships that can enhance shared learning and collaboration in pandemic response as quickly as possible.

As the coronavirus pandemic continues to evolve globally, our service adaptations and Covid-19 response offer lessons learned which ideally can be leveraged to reinvent mental health services in a post-pandemic world. First, the transition to remote and digital platforms to deliver mental health care in resource-limited settings has thus far seen fewer lapses in care than anticipated, despite challenges in network connectivity for providers and service users. Care disruptions stemming from the transition to remote services may have been offset by the new ability to connect with people living far from services, a major driver of limited care access in many settings globally. Second, the transition to remote training and care supervision within task-shared systems has gone more smoothly than anticipated. Non-specialist providers with trusting functional relationships with specialist supervisors prior to the pandemic transitioned relatively smoothly to remote clinical supervision; in some circumstances the reduction in transport time for supervisors to reach supervisees in remote areas resulted in additional time spent supervising rather than in transit. Such hybrid models could inform the development of training and supervision platforms with optimal levels of digital *v.* in-person time to enhance competency in care delivery. Third, the Covid-19 pandemic has offered an unanticipated opportunity for high-income countries to incorporate task sharing of mental health support within health systems, such as the provision of basic psychological support by non-specialist contact tracers within the Massachusetts Community Tracing Collaborative. Such examples of ‘reverse innovation’ could offer an important springboard for high-income settings to incorporate task sharing and other evidence-based innovative approaches designed to increase the reach and effectiveness of mental health services (Vigo *et al*., 2020).

Lastly, although it is well known that poverty and inequality affect mental health, the need for mental health services to address socio-economic stressors has been laid bare by the Covid-19 pandemic. Remote mental health services designed to support individuals during quarantine or isolation are useless without concomitantly addressing financial losses, reduced access to food and other basic necessities, or increased risk of violence experienced by vulnerable individuals during the pandemic. During and after the pandemic, those with preexisting mental health conditions are likely at increased risk of worsened mental health and impoverishment (Brooks *et al*., 2020). In a post-pandemic world, ‘building back better’ offers an opportunity for mental health services to move beyond individual treatment to more fully and completely acknowledge the social conditions that affect wellness and mental health, and incorporate system-based efforts to redress those conditions.

A comprehensive, integrated response to the coronavirus pandemic demands urgent attention to current mental health services and care, with bridging of lessons learned to date across high-, middle- and low-income settings, and their repackaging and delivery in community settings by non-specialists using new technologies. Collaborative alliances across systems and funders could be an essential ingredient to urgently actualizing such a critical imperative for local and global health. The widespread mental health consequences of Covid-19 can be used to reform mental health care delivery systems in a radical way. The ability of mental health systems and programs to ‘build back better’ from the coronavirus crisis will depend on the effectiveness of our collective adaptability and reaction now.
